# Molecular Detection of Multidrug Resistant *Staphylococcus aureus* Isolated from Bovine Mastitis Milk in Bangladesh

**DOI:** 10.3390/vetsci7020036

**Published:** 2020-03-30

**Authors:** Md. Salauddin, Mir Rowshan Akter, Md. Khaled Hossain, K. H. M. Nazmul Hussain Nazir, Ayman Noreddin, Mohamed E. El Zowalaty

**Affiliations:** 1 Department of Microbiology, Faculty of Veterinary and Animal Science, Hajee Mohammad Danesh Science and Technology University, Dinajpur 5200, Bangladesh; salauddin.dvm@gmail.com (M.S.); akter.rowshan@gmail.com (M.R.A.); khossainhstu@gmail.com (M.K.H.); 2 Department of Microbiology and Hygiene, Faculty of Veterinary Science, Bangladesh Agricultural University, Mymensingh 2202, Bangladesh; 3 Infectious Diseases and Anti-infective Therapy Research Group, College of Pharmacy and Sharjah Medical Research Institute, University of Sharjah, Sharjah 27272, UAE; anoreddin@sharjah.ac.ae; 4 Department of Medicine, School of Medicine, University of California, Irvine, CA 92868, USA; 5 Zoonosis Science Center, Department of Medical Biochemistry and Microbiology, Uppsala University, Uppsala SE 75185, Sweden

**Keywords:** Bovine mastitis, *Staphylococcus aureus*, 23S rRNA gene, multidrug resistance, zoonotic, methicillin-resistant, livestock, antimicrobial

## Abstract

The current study was conducted to isolate and identify multidrug-resistant *Staphylococcus aureus* (MDR-SA) from mastitis milk samples and to determine their antimicrobial susceptibility pattern. A total of 48 bovine mastitis (BM) milk samples were collected from different parts of the Rangpur division, Bangladesh. After the collection of milk samples, mastitis was confirmed using the California mastitis test. Isolation and identification of *Staphylococcus aureus* were performed using conventional cultural and biochemical tests as well as using molecular methods of PCR. Nucleotide sequence analysis of the 23S rRNA gene of *Staphylococcus aureus* was determined. The antibiogram of the isolated bacteria was conducted using the disc diffusion method. Phylogenetic analysis of 23S rRNA was done using MEGA 7, ClustalW multiple sequence alignment, and NCBI-BLAST tools, where the sequence of the isolate showed 98% to 99% identity. Antibiogram test using 15 antimicrobial agents showed that all of the *Staphylococcus aureus* isolates were classified as multidrug-resistant (MDR). It was found that the isolates were resistant to tetracycline, novobiocin, methicillin, vancomycin, and cephradine, and the isolates were sensitive to ciprofloxacin, azithromycin, norfloxacin, levofloxacin, gentamicin, and amoxicillin. The detection of MDR-SA in mastitis milk is alarming and represents a great public health concern. The findings of the present study help identify *Staphylococcus aureus* at the molecular level using 23S rRNA gene sequencing and will help select the appropriate and effective antimicrobial agent to control BM in the northern part of Bangladesh.

## 1. Introduction

Bovine mastitis (BM) is an extremely common and genuine risk to the dairy industry throughout the world. BM is difficult to control due to the multifactorial nature of the disease, of which *Staphylococcus* spp. is notoriously involved [1]. *Staphylococcus aureus* (*S. aureus*) is a potential pathogen that is responsible for intramammary infections and decreases the health status of cows, leading to huge economic losses [2]. It is the leading pathogen that frequently appears in BM, however, its identification is comparatively costly [3]. Yearly costs related to the treatment of mastitis dairy livestock are more than one million dollars in the USA [4,5,6]. Antibiotics are indiscriminately used to control BM. Antibiotic therapy is one of the most common practices for controlling BM, and antimicrobial drugs like chloramphenicol, ciprofloxacin, novobiocin, vancomycin, and tetracycline were reported to have poor effectiveness against *S. aureus* [7,8,9,10]. Several antibiotics have been used for BM treatment, however, nowadays, treatment failure is commonly reported [11]. The deliberate use of antimicrobial agents in veterinary practice is a major factor for spreading antibiotic-resistant bacterial pathogens to human populations, thus may potentially lead to public health issues [5,11]. Methicillin-resistant *Staphylococcus aureus* (MRSA) has been reported as a threat to both humans and animals [12,13,14]. Multidrug-resistant (MDR) *S. aureus* infections have been associated with high morbidly and economic losses. *S. aureus* is often found in dairy cow’s milk and has been clinically proven to be a cause of mastitis [15]. It has been reported that humans sometimes consume raw milk [16]. Nowadays, livestock-associated and community-associated MRSA have been reported and are of great public health concern [17,18,19,20]. The consumption of contaminated milk and milk products was reported to be a source of infections by *S. aureus* [21]. *S. aureus* infections in humans may lead to septicemia, pneumonia, and dermatitis [22]. There has been limited research about the detection of *S. aureus* in Bangladesh [23,24,25]. In recent years, the prevalence of BM in the Rangpur division has been reported to be gradually increasing. In the present study, 23S rRNA gene analysis was used to identify in-depth gene-specific *S. aureus* in the Rangpur division from BM milk. In addition, the study was conducted to determine the antimicrobial resistance pattern of *S. aureus* and the phylogenetic relatedness of the isolated *S. aureus* from BM, which may help control the imprudent use of antibiotics in veterinary practices for the treatment of BM and to increase public awareness.

## 2. Materials and Methods

### 2.1. Ethics Statement

The experimental procedures and protocols used in this study were approved by the Animal Ethics and Welfare Committee of the Institute of Research and Training, Hajee Mohammad Danesh Science and Technology University (approval number HSTU/RIT/2556).

### 2.2. Sample Collection and Processing

A total of 48 milk samples (10 mL) from cattle (*Bos indicus*) suffering from mastitis were collected from nine selected dairy farms of the Rangpur division of Bangladesh in September 2017 to January 2018 (Table 1). To confirm the mastitis in the cattle, the California Mastitis Test (CMT) was performed by mixing milk sample (3 mL from each quarter) and commercial CMT reagent (3 mL) using a partitioned plastic paddle, as previously described [26]. After screening the presence of mastitis by CMT tests, all milk samples were transferred to the Microbiology Laboratory, Department of Microbiology, Hajee Mohammad Danesh Science and Technology University (HSTU), for further microbiological processing.

### 2.3. Isolation and Identification of Staphylococcus spp.

All positive samples (confirmed by clinical signs and CMT test) were cultured on Nutrient Agar (HI-MEDIA^®^, Mumbai, India), Mannitol Salt Agar (MSA) (HI-MEDIA^®^, Mumbai, India) and Staphylococcus Agar No. 110 (SAN 110) (HI-MEDIA^®^, Mumbai, India) to observe colony morphology and staining characteristics, as described previously [27]. Samples were also cultured on blood agar medium (Merck, Darmstadt, Germany) enriched with defibrinated ox blood to determine the hemolytic activity of the isolates. The isolates were identified using conventional biochemical tests, as described previously [27,28,29].

### 2.4. Antimicrobial Susceptibility Study

The isolates were tested for their antibiogram using the disc diffusion method [30] using 15 antibacterial discs (HI-MEDIA^®^, Mumbai, India). The isolates were cultured on Muller Hinton Agar (HI-MEDIA^®^, Mumbai, India) according to the guidelines of the National Committee for Clinical Laboratory Standards [31]. The antibiotic discs used in the current study were gentamicin (GEN-10 µg), amoxicillin (AMX-30 µg), chloramphenicol (C-30 µg), ciprofloxacin (CIP-5 µg), bacitracin (B-10 µg), azithromycin (AZM-30 µg), erythromycin (E-15 µg), methicillin (MET-5 µg), novobiocin (NV-30 µg), vancomycin (VA-30 µg), norfloxacin (NX-10 µg), tetracycline (TE-30 µg), levofloxacin (LE-5 µg), nalidixic acid (NA-30 µg), and cephradine (CH-30 µg). The zone of inhibition was recorded in millimeters and results were interpreted as previously described [32].

### 2.5. DNA Extraction

A single pure colony of *S. aureus* strain BARC001 culture from SAN 110 was cultured overnight in Luria Bertani broth (HI-MEDIA^®^, Mumbai, India) at 37 °C. Genomic DNA of *Staphylococcus aureus* (n = 48) was extracted using a Wizard^®^ Genomic DNA Purification Kit (Promega Corporation, Madison, WI, USA) according to the manufacturer’s protocol. Extracted DNA was quantified at 260/280 nm by Nanodrop (Thermo Fisher Scientific, Dreieich, Germany) and stored at −20 °C.

### 2.6. PCR Amplifications

Reference primers (Integrated DNA Technologies (IDT), Coralville, IA, San Diego, USA) Sau 234 (F) (5′ CGATTCCCTTAGTAGCGGCG 3′) and Sau 1501 (R) (5′ CCAATCGCACGCTTCGCCTA 3′) targeting the 23S rRNA gene (Gene bank database *S. aureus*, GI no. 288516) were used to amplify the 23 rRNA gene, as previously described [33]. The primers (20 pico-moles) used in this study generated an amplicon of 1267 bp as visualized by gel electrophoresis (MGU-402T, CBS Scientific, United Kingdom). The PCR reaction volume was 25 µL and consisted of 12.5 µL 2x PCR master mix (Promega Corporation, Madison, WI, USA), 1 µL forward primer, 1 µL reverse primer, 8.5 µL nuclease-free water, and 2 µL DNA templet in each PCR tube. The PCR reaction was conducted using a thermocycler (Gene Atlas, Astec, Minamizato, Shime, Kasuya, Japan) with modifications from a previously described method [33]. The PCR reaction conditions were as follows: initial denaturation at 94 °C, 1 cycle for 2 min; denaturation at 94 °C for 45 s; annealing at 58 °C, 60.5 °C, 60.9 °C, 61.3 °C (gradient) for 60 s; extension at 72 °C for 2 min for 35 cycles; and a final extension at 72 °C for 10 min. The amplicons were visualized using 2% agarose gel (Promega Corporation, Madison, WI, USA).

### 2.7. Sequencing and Phylogenetic Analysis

The nucleotide sequence of the 23S rRNA gene was obtained by Sanger sequencing method using the ABI 3130 Genetic Analyzer (Applied Biosystems^®^, Thermo fisher scientific, Waltham, MA, USA) and sequence data were submitted to NCBI nucleotide sequence database for analysis using the BLAST tool, the ClustalW multiple sequence alignment, and neighbor-joining method [34]. The phylogenetic tree was constructed using MEGA software [35]. The evolutionary distances were computed using the maximum composite likelihood method [36].

## 3. Results

### 3.1. Isolation and Identification of MDR-SA

A total of 48 clinical mastitis milk samples were collected from dairy cow farms from different areas of the Rangpur division (Table 1). Based on cultural examinations, four bacterial species, including *Staphylococcus* spp. (100%, *n* = 48/48), *Escherichia coli* (100%, *n* = 48/48), and *Klebsiella* spp., (62.5%, *n* = 30/48), and *Streptococcus* spp. (58.3%, n = 28/48) were identified (Table 1). It was found that *S. aureus* was isolated from all mastitis milk samples obtained from dairy cows. *S. aureus* showed gray-white colonies on SAN 110 and golden yellowish colonies on MSA plates. Isolates showed complete (β) hemolysis on ox blood agar. The isolates were confirmed as *S. aureus* using different conventional biochemical tests.

**Figure 1:**
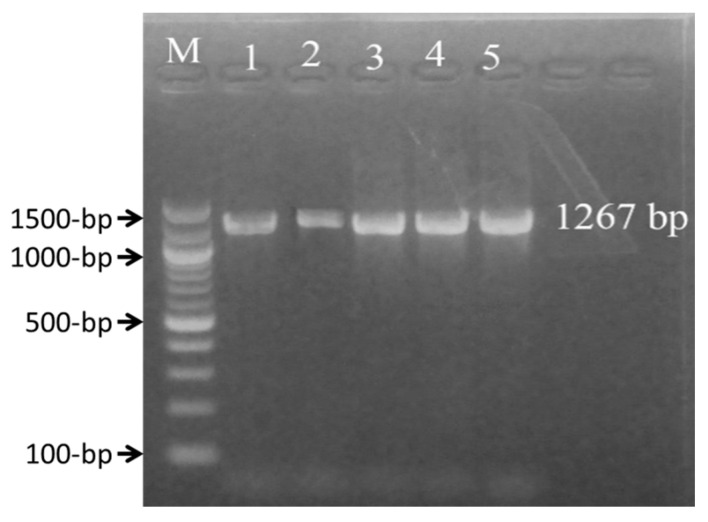
PCR amplification of 23S rRNA gene using the primers Sau 234 (F) and Sau 1501 (R) showing an amplicon size of 1267 bp. M: Marker 100 bp Plus DNA ladder.

The genomic DNA was used to confirm identity using the 23S rRNA and species-specific PCR. Isolates showed a band at 1267 bp (Figure 1). PCR products (10 pico-moles) were submitted to the National Institute of Biotechnology (NIB), Savar, Dhaka, Bangladesh for partial length sequencing of the 23S rRNA gene in both directions.

**Figure 2:**
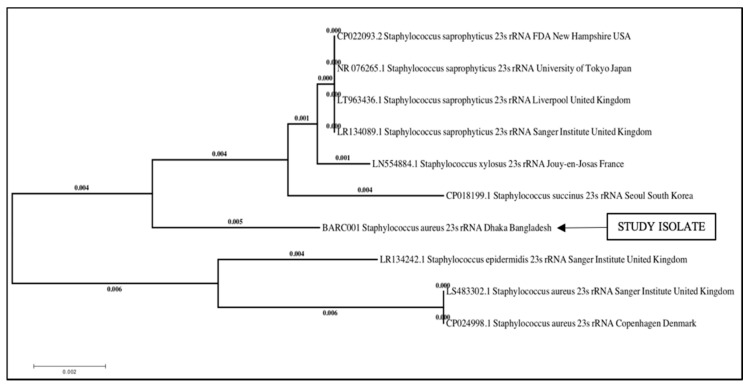
Phylogenetic tree of *Staphylococcus aureus* strain BARC001 23S rRNA gene sequence in the present study and related GenBank sequences.

BLAST analysis revealed 98% homology with NCBI GenBank data set. Phylogenetic analysis was done by ClustalW multiple sequence alignment and the neighbor-joining method (Figure 2).

The sequence analyses revealed that the *S. aureus* (n = 1) Strain BARS001 was closely related to isolates previously reported from South Korea, France, the United Kingdom, and the USA.

### 3.2. Antibiogram of S. aureus Isolates

The results of the antibiogram test of *S. aureus* isolated from mastitis milk samples against 15 antimicrobial agents revealed that all isolates tested were resistant to methicillin (MET-5 µg), novobiocin (NV-30 µg), vancomycin (VA-30 µg), tetracycline (TE-30 µg), and cephradine (CH-30 µg). On the other hand, isolates showed intermediate resistance to chloramphenicol (C-30 µg), bacitracin (B-10 µg), erythromycin (E-15 µg), and nalidixic acid (NA-30 µg). The tested isolates were sensitive to six out of the 15 tested antimicrobial agents as shown in Table 2.

## 4. Discussion

Bovine mastitis (BM) is an inflammatory disease of dairy cows caused by various opportunistic bacteria that reduce milk yield and deteriorate the quality of milk composition [37,38]. BM is a threat to dairy farms and farmers [39]. The findings of the current study showed that the prevalence of *S. aureus* in mastitis milk samples was 100% (*n* = 48). Cultural, biochemical, as well as molecular tests using the 23S rRNA gene amplification were used to identify *S. aureus*.

The presence of *S. aureus* in milk in the present study was found to be higher than previous studies [16,40,41,42,43,44]. The high prevalence rate of *S. aureus* in the present study may be explained by differences in geographical distribution, immunological status, as well as biosecurity practices of the study areas in comparison to other studies. The antibiogram showed that all the isolates were classified as MDR, similar to a previous study [45]. In the current study, levofloxacin (26 mm) and ciprofloxacin (29 mm) demonstrated the highest antibacterial activity based on the zones of inhibition, which may be recommended for use to treat BM in the Rangpur division of Bangladesh. Nevertheless, a study by Jahan [16] revealed that more than 83.33% *S. aureus* from raw milk were susceptible to ciprofloxacin. The results of the antibiogram from the current study were similar to those previously reported [16,46,47]. All isolates in the present study were classified as multidrug-resistant (MDR) [48]. The 23S rRNA sequencing was a powerful tool for the identification of *Staphylococcus aureus* isolated from bovine mastitis and its evolutionary features. In this study, 23S rRNA gene sequencing was used to identify coagulase-positive (CoPS) *S. aureus* isolates of BM at the species level. The detection of MDR-SA is alarming for human health, as it has zoonotic potential. Livestock may play an important role in transmitting the bacteria to humans through milk [49,50], which may represent a great threat to public health, farm-associated workers, and veterinarians. In recent years, the incidence of *S. aureus* has been increasing, and health care-associated MRSA and livestock-associated MRSA have commonly been reported, which has a major impact on public health [51,52]. The phylogenetic analysis showed that the MDR-SA isolate is closely related to those previously reported from other countries and regions [9,16,17].

## 5. Conclusions

Livestock associated CoPS -SA, which is MDR, is notorious for its appearance in the Rangpur division, causing BM. Current research suggests that it is essential to stop the deliberate use of antibiotics to prevent MDR-SA. Proper hygienic practices and the maintenance of biosecurity measures are two of the most important steps to reduce infections. The findings of the present study help determine the evolutionary feature of *S. aureus* in the Rangpur division, and the antibiogram results help select the appropriate antimicrobial agents for the treatment of infected animals. The isolated *S. aureus* may be used for the future development of a mastitis vaccine using local isolates as a prophylactic measure in livestock.

## Figures and Tables

**Table 1:** Sample collection and frequency of bacterial isolates in the milk samples.

Samples Collection Area	Number of Samples	Samples Positive for *E. coli* (%)	Samples Positive for *Staphylococcus* spp. (%)	Samples Positive for *Streptococcus* spp. (%)	Samples Positive for *Klebsiella* spp. (%)
Chirir bandar	12	100	100	58.34	50
Sadar Livestock office Dinajpur	8	100	100	62.5	62.5
Nandigram, Birganj	5	100	100	60	60
Birol, Dinajpur	10	100	100	70	60
Sadullahpur, Gaibandha	2	100	100	100	50
Kornai, Baserhat	2	100	100	-	100
Thakurgaon, Sadar	2	100	100	100	50
Thakurgaon, Haripur road	4	100	100	50	75
Birganj Livestock office	3	100	100	66.67	100
Total	48	48	48	28	30

**Table 2:** Antibiogram study of *Staphylococcus aureus* isolates in the current study.

		Antibacterial Agents (μg/disc)														
Isolates (*n* = 48)	Diameter of Zone of Inhibition (mm)	AMX (30)	C (30)	NX (10)	TE (30)	GEN (10)	B (10)	AZM (30)	E (15)	NV (30)	MET (5)	VA (30)	LE (5)	CIP (5)	CH (25)	NA (30)
*Staphylococcus aureus*	S	15 mm		19 mm		21 mm		23 mm					26 mm	29 mm		
	I		20 mm				11 mm		17 mm							24 mm
	R				0					0	0	0			0	

GEN: gentamicin; AMX: amoxicillin; C: chloramphenicol; CIP: ciprofloxacin; AZM: azithromycin; E: erythromycin; VA: vancomycin; NX: norfloxacin; TE: tetracycline; B: bacitracin; NV: novobiocin; MET: methicillin; LE: levofloxacin; CH: cephradine; NA: nalidixic acid; 0: no zone of inhibition; S: sensitive; I: intermediate; R: resistant.

## Data Availability

The 23S rRNA sequence of *Stapylococcus aureus* strain BARC001 has been deposited at the National Center for Biotechnology Information (NCBI), U.S. National Library of Medicine (NLM) (Bethesda MD, USA) under the BioProject number PRJNA613919 (https://www.ncbi.nlm.nih.gov/bioproject/?term=PRJNA613919) (BioSample accession number SAMN14421930 (https://www.ncbi.nlm.nih.gov/biosample/?term=SAMN14421930) and GenBank accession number (https://www.ncbi.nlm.nih.gov/nuccore/MT232660)).
